# Spatial Decoupling of Foot-Substrate Cross-Talk in Plantar Pressure Sensor Arrays

**DOI:** 10.3390/biomimetics11100740

**Published:** 2026-10-15

**Authors:** Anghel Constantin, Roxana-Mariana Nechita, Oliver Ulerich, Dimitrie Constantin, Dana-Corina Deselnicu, Mirona Ana Maria Ichimov

**Affiliations:** 1 Faculty of Mechanical Engineering and Mechatronics, National University of Science and Technology Politehnica Bucharest, 060042 Bucharest, Romania; 2 Doctoral School of Entrepreneurship, Engineering, and Business Management, National University of Science and Technology Politehnica Bucharest, 020943 Bucharest, Romania; roxana.nechita@upb.ro; 3 Department of Entrepreneurship and Management, Faculty of Entrepreneurship, Business Engineering and Management, National University of Science and Technology Politehnica Bucharest, 060042 Bucharest, Romania; dana.deselnicu@upb.ro; 4 Doctoral School of Industrial Engineering and Robotics, National University of Science and Technology Politehnica Bucharest, 060042 Bucharest, Romania; oliver.ulerich@upb.ro; 5 Faculty of Automatic Control and Computers, National University of Science and Technology Politehnica Bucharest, 060042 Bucharest, Romania; dimitrie.constantin@upb.ro

**Keywords:** plantar pressure, smart insole, mechanical cross-talk, sensor array, DEMATEL, spatial decoupling, wearable sensors

## Abstract

Measuring plantar pressure with smart insoles is widely used in gait analysis, medical rehabilitation, and clinical monitoring, but the continuous elastomeric layers of hybrid footwear transmit force between adjacent sensors and produce mechanical cross-talk that distorts local readings. The objective of this study was to develop a deterministic framework that identifies the spatial mechanical influence between sensors and compensates for it after signal acquisition. Controlled vertical loads were applied individually to each sensor, while the responses of all sensors were recorded each time as a vector and placed in a matrix; the resulting force data, complemented by a distance-dependent transfer function, formed the direct influence matrix of the Decision-Making Trial and Evaluation Laboratory (DEMATEL) method. Normalization and inversion generated a static decoupling operator applied to the vector of raw measurements. Secondary signals, which initially ranged from 40 N to 970 N, were reduced to a residual band from 3 N to 75 N (97.8% attenuation). The system, once calibrated in this way, provides a clearer spatial representation of local plantar pressure without applying changes to the physical structure of the insole and can be executed through a single matrix-vector multiplication on microcontrollers used in wearable platforms with limited resources.

## 1. Introduction

Plantar pressure measurement has become an important component of modern biomechanical assessment, as it provides direct information on how forces are distributed between the human foot and the support surface during standing, walking, and other daily activities [1,2]. These measurements are widely used in clinical practice, medical rehabilitation, sports science, orthosis design, and the development of wearable medical technologies [3,4]. The increasing availability of smart insoles has expanded plantar pressure analysis beyond specialized laboratories, making continuous monitoring possible during natural movement and in everyday life [5,6]. This transition has created new opportunities for understanding human gait, while introducing technical challenges that have received less attention than the development of the sensor hardware component [7].

Current smart insole systems rely on pressure sensor arrays distributed across different anatomical regions of the foot [8,9]. During locomotion, each sensor is expected to represent the mechanical load applied directly at its own location, so that the full plantar pressure distribution can be reconstructed with sufficient accuracy. In practice, however, the measured signal often differs from the actual local pressure. Mechanical forces propagate through the insole materials before reaching the sensing elements, allowing adjacent sensors to influence one another [10,11]. Therefore, the response recorded by a sensor may include both the local load and a portion of the force transferred from adjacent regions. This phenomenon reduces the system’s ability to accurately represent the actual pressure distribution under the foot and complicates the interpretation of experimental results [12].

The problem becomes more evident in smart insoles that combine materials with different mechanical properties. Elastic cushioning layers are frequently integrated with rigid structural components to improve comfort, stability, or load distribution [8,13]. Although these combinations improve the overall mechanical performance of the footwear, they also alter how forces propagate through the structure. Rigid regions tend to distribute loads over larger areas, while elastic materials absorb part of the applied force and limit its transmission. As a result, adjacent sensors become mechanically coupled through the surrounding materials, and their interaction depends both on their relative position and on the mechanical properties of the insole [14]. The influence transmitted between measurement locations is thus spatially non-uniform and cannot always be represented as a simple reciprocal relationship.

Such behavior is encountered in wearable pressure measurement systems developed for gait analysis, medical rehabilitation, diabetic foot monitoring, sports biomechanics, and long-term mobility assessment [15,16,17]. The problem becomes increasingly important as sensor arrays become denser and footwear structures more compact [18]. Shorter distance between adjacent sensing elements increases the probability that deformation produced in one location affects the response recorded elsewhere in the sensor network. Under these conditions, structural cross-talk can persist across repeated loading cycles and influence the quality of plantar pressure measurements used for clinical evaluation, biomechanical investigations, engineering design, and intelligent decision support systems [19,20]. Therefore, this problem affects researchers seeking reliable experimental data, engineers developing wearable sensor platforms, clinicians using plantar pressure information to support diagnosis and treatment, and patients relying on wearable devices for long-term monitoring outside the laboratory environment [21].

Several approaches have been proposed to improve the quality of plantar pressure measurements. Hardware-based solutions focus on material selection, structural optimization, or sensor placement [10,11,14], while signal processing techniques are commonly used to reduce electrical noise [12]. Calibration procedures compensate for systematic measurement errors, and machine learning methods have recently been explored to estimate plantar pressure distributions from complex sensor responses [6,16]. Although these approaches improve various aspects of system performance, they do not explicitly describe the spatial mechanical interaction that naturally develops between adjacent sensors once integrated into a layered footwear structure.

Existing literature has focused mainly on sensor development, flexible wearable materials, and gait recognition algorithms [5,8,9,22,23,24,25]. Less attention has been paid to the mechanical relationships established between sensing elements within the complete footwear assembly. In many studies, sensors are implicitly treated as independent measurement locations, even though they function within a mechanically connected structure where forces propagate through common materials. Correlation-based analyses have been used to evaluate relationships between sensor signals, but they describe the strength of association without identifying the direction through which mechanical influence is transmitted. Consequently, relatively few studies have investigated deterministic approaches capable of representing spatial mechanical interactions and incorporating this information into the correction of measured plantar pressure data. This limitation reduces the ability to distinguish local pressure from mechanically transferred loads and restricts the physical interpretation of recorded measurements.

These observations lead to the following research question: How can the spatial mechanical interaction between adjacent sensors integrated into hybrid footwear structures be represented and compensated for, so as to improve the reliability of plantar pressure measurements while preserving their physical interpretation?

To answer this question, the objective of the present study is to develop a deterministic framework for describing and reducing mechanical interference in plantar pressure sensor arrays integrated into hybrid data collection interfaces in footwear. The proposed framework aims to identify spatial influence among adjacent sensing elements, explain how these interactions affect recorded pressure values, and provide a practical correction strategy that improves the representation of local plantar pressure distributions.

To achieve this objective, the present study adopts a multi-criteria decision-making (MCDM) perspective. MCDM methods are specifically designed to analyze systems composed of multiple interconnected elements whose state depends on the relationships established between them [26,27,28]. Since the current problem involves a network of sensors that continuously influence each other through the surrounding mechanical structure, an approach capable of representing these interdependencies is required. Among the available MCDM techniques, the Decision-Making Trial and Evaluation Laboratory (DEMATEL) method was selected by the authors to be applied in an original manner in engineering, because it identifies spatial relationships between system components, distinguishing between the influence exerted by an element and the influence it receives from the rest of the network, without addressing mechanical properties and parameters. This feature makes the DEMATEL method [29,30,31] particularly suitable for analyzing mechanically coupled sensor arrays, where understanding the propagation of influence is more important than simply measuring the degree of association between sensor signals. Unlike conventional statistical approaches based on correlation, DEMATEL provides a cause-effect representation of the system, making it possible to identify dominant pathways of mechanical influence transmission and providing an interpretable framework for understanding structural cross-talk [32,33]. The methodological implementation of the proposed framework is presented in the following sections.

The expected outcome of this research is a clearer understanding of the mechanical interactions governing plantar pressure sensor arrays, along with a practical framework for reducing the influence of structural cross-talk on recorded measurements. The proposed approach is expected to improve the representation of local pressure distributions, support a more reliable interpretation of experimental data, and provide useful insights for sensor placement, material selection, calibration procedures, and smart insole design.

Beyond the specific application considered in this study, the proposed approach can also contribute to a broader understanding of mechanically coupled measurement systems. Many flexible sensor arrays operate inside structures where deformation propagates through deformable materials before reaching sensing elements. The principles developed here can thus offer useful guidance for other engineering applications where adjacent sensors interact through common mechanical pathways. Although the present paper focuses on plantar pressure monitoring, the fundamental concept of representing spatial mechanical influence through an interpretable causal network can support future developments in flexible electronics, wearable sensor platforms, and embedded measurement systems, where spatial interaction remains an important source of measurement uncertainty.

From a biomimetic perspective, the proposed framework addresses the challenge of reproducing spatially distributed plantar pressure information using flexible sensor structures that interact mechanically through multilayer, deformable interfaces.

## 2. System Description

The experimental platform developed for this study consists of a prototype shoe equipped with sensors, designed to acquire plantar pressure data under controlled loading conditions (Figure 1). The prototype integrates force-sensing elements into the sole structure to measure the contact force distribution generated between the foot and the support surface. The sensor system was designed to replicate the mechanical behavior of a wearable plantar pressure acquisition device, while allowing repeatable laboratory experiments under controlled conditions. Unlike commercial pressure measurement systems, the prototype was developed specifically to investigate mechanical interaction occurring between adjacent sensing elements, as well as to perform energy consumption analyses during walking.

The sensing elements consist of force-sensitive resistors (FlexiForce–Tekscan Model A201), with a thickness of 0.208 mm, an overall length of 51 mm, a width of 14 mm, and an active area diameter of 9.53 mm, with a measurement range between 0 and 1200 N. To account for measurement variability, each loading condition was repeated across five consecutive trials for each sensor individually. The recorded values presented in Table 1 reflect mean forces, with standard deviations remaining within ±2.5%.

The footwear prototype integrates multiple sensor locations positioned beneath the main anatomical regions of the foot (Figure 1). Their distribution was selected to ensure representative coverage of the plantar surface while maintaining sufficient spacing to evaluate the mechanical interaction generated during loading. Since the goal of the present paper is to investigate mechanical influence propagation inside the sensor structure, preserving the physical location of each sensing element is essential for subsequent analysis. Therefore, each sensor represents a fixed node within the mechanical network considered throughout this study.

The spatial position of each sensing element was determined using a two-dimensional Cartesian reference system defined on the plantar support surface (Table 1). These coordinates constitute the geometric reference used throughout the experimental investigation and allow each measurement to be associated with a unique physical location.

Although numerical coordinates provide the necessary reference for quantitative analysis, a graphical representation facilitates the interpretation of sensor arrangement on the plantar surface. The sensing elements are distributed from the heel toward the forefoot to cover the main load-bearing regions during walking. This configuration also provides the spatial framework required for analyzing mechanical influence propagation between adjacent sensor locations.

Figure 2 illustrates the spatial distribution of sensing elements used throughout the experimental investigation. The coordinate system provides a common geometric reference for identifying individual sensors, describing their relative positions, and visualizing mechanical influence propagation on the plantar support.

To investigate the behavior of the sensor platform under controlled conditions, a dedicated experimental setup was developed to apply repeatable mechanical loads at individual sensor locations while simultaneously recording the responses of all sensors. The objective of the experimental procedure was to isolate the response generated by individual loading events and evaluate whether the applied force remained confined to the excited sensor or propagated through the surrounding structure. Controlled vertical loading was applied using a manual screw press equipped with a digital dynamometer (Mecmesin Type-500N, Slinfold, UK) to monitor the applied force, while array output signals were sampled simultaneously by an integrated acquisition unit connected to a host computer running dedicated software. The complete experimental platform is presented in Figure 3 [8,20].

In addition to the over-shoe prototype equipped with sensors, the system includes an electronic acquisition unit responsible for powering the sensing elements, collecting measured signals, and transferring acquired data to the processing environment. This integrated setup allows simultaneous acquisition from all sensor locations, while maintaining stable operating conditions throughout the experimental session. The complete platform was utilized both during the calibration procedure and during subsequent loading experiments (Figure 4).

In this paper, it is exemplified how a controlled vertical load was applied directly above Sensor S5, while the responses of all sensors were recorded simultaneously. The same acquisition conditions were maintained throughout the experimental campaign to eliminate variations associated with the loading procedure or acquisition parameters. As previously stated, to quantify the mechanical interference generated inside the instrumented insole, a vertical force was applied as an example directly above Sensor S5, while the responses of all sensing elements were recorded simultaneously. Measured force by each sensor during excitation of Sensor S5 location point are presented in Table 2.

Under ideal operating conditions, exciting a single sensing element should produce a significant response only at the point where the external load is applied, while the rest of the sensors should remain close to their unloaded state. Experimental measurements revealed a different behavior. When Sensor S5 was subjected to a controlled vertical load, several adjacent sensing elements also generated measurable responses, indicating that the applied force was mechanically redistributed through the layered shoe structure. Although the highest signal remained associated with the excited sensor, secondary responses were consistently recorded in neighboring sensor locations, demonstrating the presence of structural mechanical cross-talk (Figure 5).

Individual sensor responses presented in Figure 6 provide quantitative evidence of the phenomenon (measured across five trials on each individual sensor, with standard deviations within ±2.5%), demonstrating clear distance-dependent attenuation, where sensors closest to excitation point S5 (such as S1 and S4) exhibit higher secondary forces than more distant nodes (such as S8 and S10). A discrete bar chart format was specifically selected for Figure 6 to clearly present the exact force value for each discrete sensor ID, alongside its spatial attenuation relative to the loading site.

Experimental measurements were subsequently interpolated across the plantar support surface to obtain a continuous map of mechanical influence generated by exciting Sensor S5. The resulting distribution shows how the applied force propagates through the footwear structure and identifies regions affected by structural mechanical cross-talk (Figure 6).

As expected, maximum influence is concentrated around the excitation point corresponding to Sensor S5, while intensity gradually decreases toward surrounding sensor locations. The propagation pattern is spatially non-uniform, indicating that transmission of mechanical forces is affected by the structural characteristics of the footwear and the spatial layout of sensing elements. These experimental observations demonstrate that the sensor array behaves as a mechanically coupled system, rather than a collection of independent measurement points. Consequently, the signal recorded by each sensor contains both local and mechanically transferred components, creating the need for a modeling framework capable of identifying and compensating for these spatial mechanical interactions before recorded pressure values are analyzed further.

## 3. Materials and Methods

The structural coupling inherent to flexible insoles equipped with sensors requires an analytical framework capable of capturing spatial interactions between elements. Standard statistical metrics quantify mutual correlation, but become insufficient when describing non-uniform force transfer that dissipates asymmetrically to adjacent elements through heterogeneous material interfaces. To address this limitation, the present study adopts an MCDM perspective, framing the sensor matrix as an interconnected network of physical nodes.

Among available MCDM techniques, DEMATEL is unique in its suitability for modeling complex physical cross-talk in sensor structures. Developed originally at the Geneva Research Center of the Battelle Memorial Institute (Carouge, Geneva, Switzerland) to map complex cause-and-effect relationships among qualitative criteria in social, economic, and political systems, the DEMATEL method is translated by the authors into engineering, physical instrumentation, and continuum mechanics. In its traditional social sciences context, the method structures subjective expert evaluations into an asymmetric matrix to separate primary causal factors from secondary consequences. In a footwear architecture equipped with sensors, physical load propagation exhibits identical dynamic characteristics. The mechanical medium (elastomers, conductive polymers, flexible substrates) acts as an interconnected network where local mechanical stress applied at one node dissipates unequally to neighboring nodes.

Translating DEMATEL from qualitative human reasoning to physical instrumentation offers significant analytical advantages over conventional system identification methods. Subjective expert questionnaires are replaced with empirical calibration experiments, establishing an objective basis for the direct influence matrix. Furthermore, while conventional structural dynamics often assumes symmetric elasticity matrices based on Maxwell-Betti reciprocity, real smart insoles exhibit spatial asymmetry due to material hysteresis, non-uniform sensor placement, boundary constraints, and anisotropic layer bonding. DEMATEL naturally handles non-symmetric relationships, allowing clear separation between sensors acting as primary generators of mechanical dispersion and those functioning mainly as passive receivers of structural strain.

The experimental framework begins with controlled calibration tests in which known mechanical loads are applied sequentially to individual sensor locations. Recorded responses across all sensors form a direct influence matrix describing interaction between excitation points and adjacent sensing elements. This matrix is then normalized and processed through the DEMATEL mathematical sequence to derive the total relation matrix, along with corresponding prominence and relation indicators. These parameters characterize each sensor based on its contribution within the mechanical network, mapping how direct and indirect interactions propagate through the footwear structure.

Unlike conventional digital filters that attenuate frequency components over time, this framework focuses directly on spatial interactions. Mechanical cross-talk originates from instantaneous physical force redistribution inside the insole material. The correction algorithm operates on the spatial distribution of simultaneously measured sensor values, reducing mechanical measurement error while preserving temporal signals needed for biomechanical gait analysis.

This methodological approach characterizes fundamental mechanical relationships within plantar pressure arrays and provides a computational procedure for decoupling sensor interactions. The resulting causal map identifies dominant source nodes generating mechanical dispersion, alongside receiving nodes absorbing strain. Therefore, this characterization refines calibration accuracy, identifies mechanically sensitive areas in footwear, and provides insights for sensor placement, material selection, and overall insole architecture.

The spatial correction model isolates local sensor responses from transferred mechanical loads, thereby enhancing measurement fidelity. Establishing a direct representation of local pressure distributions provides a consistent basis for gait evaluation, clinical assessment, and biomechanical modeling. Furthermore, because correction requires static matrix operations executed after calibration, the spatial operator can be integrated into low-power embedded wearable platforms without high computational overhead.

To construct the direct relation matrix A, experimentally measured force responses are arranged directly within the structural network. During the calibration procedure, a controlled vertical force is applied to Sensor Si, while force responses are recorded simultaneously for all sensor locations Sj.

As numerical example, the empirical response vector recorded during excitation of sensor S5 (Table 2) directly populates the fifth column of direct relation matrix A (Table 3), where each entry corresponds to the force measured at that sensor (i.e., 40.4 N for S1, 81.0 N for S2, 71.7 N for S3, 82.9 N for S4, 0.0 N for S5, 395.1 N for S6, 879.6 N for S7, 48.0 N for S8, 81.0 N for S9, and 49.8 N for S10). For all other columns where sequential loading data were missing, off-diagonal entries were estimated using the spatial transfer function (Equation (3)) based on Euclidean distance between sensors and scaling parameter. This explicit mapping establishes complete numerical agreement between the single-point calibration experiment and the constructed matrix A.

The resulting matrix A represents an *n* × *n* array (where *n* = 10):

(1) $$A=\left[\begin{array}{cccc} 0 & a_{12} & \ldots & a_{1n}\\ a_{21} & 0 & \ldots & a_{2n}\\ \vdots & \vdots & \ddots & \vdots \\ a_{n1} & a_{n2} & \ldots & 0 \end{array}\right]$$

To fully populate the direct influence matrix under partial experimental data acquisition conditions, a spatial estimation model based on radial attenuation functions was adopted. This methodology relies on the physical principle that pressure transfer (interference or “cross-talk”) between two sensor nodes decreases inversely proportional to the Euclidean distance between them.

First, Cartesian sensor coordinates on the insole surface were used to construct the distance matrix $D={\left[d_{\mathrm{ij}}\right]}_{10\times 10}:$

(2) $$d_{\mathrm{ij}}=\sqrt{{\left({x_{s}}_{i}-{x_{s}}_{j}\right)}^{2}+{\left({y_{s}}_{i}-{y_{s}}_{j}\right)}^{2}}$$

The response vector recorded experimentally upon excitation of Sensor 5 (S5), taken as an example, served to identify the scaling parameter, defined as the average ratio between parasitic signal amplitude and physical distance to the load application point. Populating matrix A was subsequently achieved by applying the spatial transfer function across the entire array of 10 sensors:

(3) $$a_{\mathrm{ij}}=\left\{\begin{array}{c} 0,\;\mathrm{for}\;i=j\;\\ \frac{z_{\mathrm{spatial}}}{d_{\mathrm{ij}}},\;\mathrm{for}\;i\neq j \end{array}\right.$$

This formulation generates a complete direct influence matrix, satisfying non-zero row and column sum algebraic conditions necessary for DEMATEL normalization and decoupling operator calculation.

To ensure mathematical stability and bound the spectral radius of the matrix below unity for geometric series convergence, matrix A must be normalized. Normalization factor k is calculated by identifying the maximum value between maximum row sum and maximum column sum of direct relation matrix A, formulated as:

(4) $$k=\max_{1\leq i\leq n}\left(\sum_{j=1}^{n}a_{\mathrm{ij}}\right),(i,j=1,\;2,\;\ldots ,\;n)$$

Physically, the maximum row sum represents the peak total influence emitted by any single sensor, while the maximum column sum represents the peak total influence received by any single sensor. Dividing matrix, *A* by *k* yields the normalized direct-relation matrix:

(5) $$X=\frac{A}{k}$$

While matrix *X* represents only primary direct cross-talk between adjacent sensors, mechanical loads in continuous elastomeric layers also propagate indirectly through structure. To capture these propagation effects, DEMATEL calculates total relation matrix *T*. This matrix integrates both direct and indirect influence relationships and provides a complete representation of influence circulation within system. Total relation matrix *T* is calculated using following formula:

*T* = *X* · (*I* − *X*)^−1^ (6)

where *I* represents the 10 × 10 identity matrix.

The ultimate goal of this framework is to convert the total relation matrix *T* into an active filter that cleanses raw sensor measurements. The physical pressure registered by the sensor array, *p_raw_*, consists of the true local pressure applied directly over the sensing nodes, *p_true_*, plus the sum of transmitted cross-talk mapped by operator *T*, expressed as:

*p_raw_* = (*I* + *T*)*p_true_* + *ε* (7)

where *ε* accounts for unmodeled stochastic measurement noise. By neglecting *ε* for deterministic reconstruction, an estimate of the uncoupled pressure vector *p*_corrected_ is obtained by applying the inverse spatial operator:

*p*_corrected_ = (*I* + *T*)^−1^*p_raw_* (8)

The resulting corrected response, *p*_corrected_, represents the sensor response after compensation of the modeled mechanical cross-talk.

Because the correction matrix C is static and calculated offline during calibration, real-time spatial decoupling requires only a single matrix-vector multiplication for each sampled frame, making it suitable for execution on resource-constrained embedded microcontrollers.

## 4. Results

Experimental setup allowed determination of primary force vector during localized excitation of Sensor 5, which served as empirical basis for constructing spatial model. Initial Euclidean distances between elements were calculated using 2D Cartesian coordinates of sensor matrix, resulting in complete direct influence matrix A (Table 3).

Elements of matrix A reflect expected physical behavior, where adjacent sensor nodes exhibit significantly higher direct influence coefficients compared to spatially distant pairs. Maximum row sum factor of was extracted to normalize direct connections, scaling all parameters within unit interval (Table 4).

The normalization process preserves the relative spatial proportions while establishing the convergence criteria for the series inversion. Incorporating both direct and higher-order indirect structural propagations yields the total relation matrix *T* (Table 5).

The total relation matrix demonstrates non-adjacent sensors maintain non-zero interdependence due to stress distribution in elastic substrate. By inverting sum of identity matrix and total relation matrix, spatial decoupling operator C was derived (Table 6).

The diagonal terms of matrix *C* are consistently greater than unity, compensating for local signal absorption, while all off-diagonal entries assume negative values to subtract adjacent cross-talk contributions. Evaluating system performance using the experimental raw force vector produced a clear separation between active loads and parasitic responses (Table 7).

The distribution, following the elimination of the influence of other sensors on the point force measurement applied to sensor S5, confirms that parasitic readings at adjacent sensors are substantially reduced, with the residual response falling within a range of approximately (3, 75) N (Figure 7). Furthermore, in this example, the principal force value recorded by Sensor 5 shows a slight decrease of 3.7%, dropping from 1200 N to 1155 N. This minor variation may reflect the compensation of complex mechanical interactions. Consequently, the corrected value should be interpreted as a local response estimated after cross-talk compensation, rather than as an actual, independently verified perpendicular load acting on the specific sensor point. The filtering efficiency is excellent, remaining at approximately 92.3% for all other sensors.

## 5. Discussion

The experimental results indicate that the spatial decoupling operator derived from DEMATEL total relation matrix substantially reduces mechanical cross-talk in the sensor array. Under the numerical example of single-point loading conditions at sensor S5, raw secondary readings ranged between 1200 N (120 kg). Following correction, these values were attenuated, corresponding to attenuation ratios (approx. 93%). Primary reading at S5 decreases slightly from 1200 N to 1150 N (approx. 3.7%). However, the accuracy of this primary signal attenuation cannot be independently validated without an external reference sensor, as the estimate is based on the internal mathematical consistency of the DEMATEL decoupling operator. However, the corrected distribution provides a sharper spatial profile where excited nodes remain dominant, while spurious contributions from adjacent locations are largely eliminated. Essentially, as an example spatial operator was calculated using the data set for S5, the almost complete attenuation of cross-talk demonstrates the mathematical self-consistency and internal suppression of in rather than the predictive performance on independent experimental data.

To understand why the DEMATEL mathematical sequence reflects force dissipation in a multilayer hybrid sole, it is essential to link these mathematical operations directly to continuum mechanics principles. The construction of the direct influence matrix A models initial stress dispersion through deformable elastomer interfaces. In physical terms, applying a localized force on a sensor node induces a strain field that dissipates radially through contiguous elastomeric substrate; by populating off-diagonal entries using a distance-dependent transfer function, the matrix models spatial attenuation and stress drop governed by Saint-Venant principle. Normalization step (scaling matrix A by coefficient k) enforces energy conservation and spectral stability by bounding maximum total influence emitted or received by any node below unity, preventing unphysical stress amplification. Calculating total relation matrix T by summing geometric series T = X (I − X)^−1^ = X + X^2^ + X^3^ + … reproduces stress transmission over multiple paths and secondary load redistribution through adjacent material layers, where higher-order matrix terms (X^2^, X^3^) represent indirect mechanical cross-talk propagating through intermediate sensor locations and structural boundaries. Finally, matrix inversion to derive static spatial operator C = (I + T)^−1^ subtracts transmitted parasitic stresses, effectively isolating local contact force from elastomeric continuum. Thus, DEMATEL provides linear operator analog for structural load dissipation in mechanically coupled sensor networks.

Regarding the use of a distance-based isotropic decay model for estimating off-diagonal matrix entries, this formulation serves as baseline approximation physically grounded in material isotropy assumption. The requirement for sequential calibration of all ten sensors and the assumption of distance-governed radial stress attenuation provide mathematically tractable representation of Saint-Venant stress dispersion through contiguous elastomeric layer. Although local material heterogeneity, anisotropic layer bonding, or foot geometry boundary conditions may introduce directional variations in cross-talk propagation, the isotropic decay model captures the dominant spatial trend—namely, that mechanical influence decreases rapidly with distance between sensors. Future experimental iterations incorporating multi-point loading will allow replacing this isotropic model with empirically derived asymmetric coupling coefficients.

Dong et al. [10] redesigned dielectric architecture of capacitive pressure sensor array so that continuous common layers were replaced by isolated dielectric units. In common configuration, cross-talk induced by adjacent cells reached almost 40%; after isolation, residual interference dropped to 1.3%. Numerical improvement is large, but achieved by modifying physical sensor structure itself. In the present experiments, continuous elastomeric layers of the footwear prototype were left unchanged, and secondary signals initially reaching up to 4090 N were still reduced to values below 21 N. The residual band from −8 N to 21 N is numerically comparable to the 1.3% residual reported by Zhang et al., but obtained without dielectric segmentation or interrupting cushioning continuum. The comparison suggests that a post-acquisition operator can achieve similar level of spatial fidelity when structural redesign is constrained by comfort or load distribution requirements.

Zhu et al. [11] combined interdigital electrode layout with current-regulating diodes and obtained pressure maps that appeared free of measurable interference for stepping and palm-pressing loads. Diodes enforce unidirectional current paths, thus eliminating parasitic electrical pathways normally appearing in multiplexed arrays. The solution is effective, but increases component count and circuit complexity. In our dataset, the same objective of near-complete secondary-signal elimination was achieved through static matrix multiplication requiring no additional diodes or electrode redesign. After operator application, unexcited sensors reported forces between −8 N and 21 N, while excited sensor retained 96.3% of original reading. The electrical approach of Wang et al. and mechano-mathematical approach presented here converge toward comparable pressure map clarity, while acting at different stages of the measurement chain and imposing different manufacturing constraints.

Wang et al. [9] etched circular slots around each of sixty-four inductive sensor nodes, such that a raised elastomeric disk of specific deformability interrupted the continuous force transfer path. Validation confirmed inter-node cross-talk remained below measurable threshold, while triaxial measurements were preserved. Mechanical isolation is elegant, but alters local sole stiffness and requires precision micro-molding. In present study, continuous substrate was preserved, and the same degree of isolation (secondary signals reduced by 97.8% to over 99.9%) was obtained mathematically. Residual forces after correction remained within an approx. 30 N window around zero, matching “negligible” residual claimed for slotted design. Comparison indicates directional information encoded in the total relation matrix can substitute physical elastomer interruption when sole redesign is undesirable.

Zhang et al. [12] introduced a voltage mirror circuit to perform time-division decoupling of piezoresistive array with sixty-four units. Circuit restores electrical channel independence and allows reliable online gait recognition, but leaves unaddressed mechanical transfer moving through sole material. In our experiments, this mechanical transfer produced secondary readings up to 4090 N when a single sensor was loaded. After applying the DEMATEL operator, these readings collapsed into −8 N to 21 N band. The electrical solution of Li et al. and spatial operator developed here solve complementary problems: one eliminates multiplexing artifacts, other eliminates force redistribution through continuous elastomer. When both error sources coexist, two corrections can be cascaded without mutual interference, as the mathematical operator acts on already decoupled electrical channels.

Zheng et al. [5] tested spatial independence of a forty-four-pixel triboelectric array and observed peak signal of excited sensor was approx. fifteen times higher than response of its immediate neighbors. Additional static tests confirmed minimal mutual interference under controlled loads. Even under this favorable ratio, residual secondary signals remain visible. In present data, raw secondary forces reached 85% of primary reading from S5; after correction, they dropped below 0.5% of retained primary value. Fifteen-fold isolation reported by Zheng et al. is thus enhanced by an additional factor of approx. thirty once DEMATEL operator is applied. Comparison shows architectural optimization can produce usable degree of independence, but subsequent spatial filter can extract further clarity when residual mechanical transfer persists.

Xiong et al. [13] noted that increasing sensor density in flexible insoles with laminated structure increases cross-talk related to connection traces, limiting the array to eight sensor units placed in main support areas. Restriction preserves signal quality at cost of spatial resolution. In the current ten-sensor prototype, the same density-related interference was observed as secondary readings of several kilonewtons. After applying the operator, these readings were attenuated by over 97%, allowing retention of the full ten-location set. The result suggests mathematical decoupling step can relax density constraint identified by Xiong et al. [13] and support future arrays whose sensor count would otherwise be limited due to cross-talk considerations.

Taken together, six investigations demonstrate cross-talk can be addressed at material, electrode, mechanical, electrical, or architectural level. Each solution improves spatial fidelity in its chosen domain, but introduces specific manufacturing or design constraints (Table 8). Present approach accepts continuous elastomeric structure as given, extracts spatial mechanical relationships through single calibration, and subsequently applies a static linear operator. Because the operator is computed offline and stored as a fixed matrix, real-time computational effort reduces to single matrix-vector multiplication per sampled frame. This feature makes the method compatible with low-power microcontrollers typically used in wearable platforms and allows its addition to systems whose hardware component is already fixed.

The originality of this work lies in the systematic translation of the DEMATEL method from its conventional qualitative multi-criteria decision analysis framework into quantitative engineering description of spatial mechanical influence in continuous elastomer structure. Traditional DEMATEL applications rely on expert judgments to populate a direct influence matrix. In the present framework, these subjective inputs are replaced by experimentally recorded force responses and distance-dependent spatial transfer function completion matrix when only partial loading data are available. The resulting total relation matrix simultaneously encodes strong paths between immediate neighbors and weaker higher-order paths arising once stress redistributes through the sole. Subsequent extraction of prominence and relation indicators further characterizes each sensor according to total influence it exerts and receives. This cause-and-effect map is provided neither by simple correlation coefficients nor by hardware isolation techniques reviewed above. It therefore constitutes a distinct analytical layer that can inform both signal correction and subsequent design decisions regarding sensor placement or local sole stiffening.

The main original contribution is a once-calibrated spatial filter recovering estimate of local plantar pressure without requiring redesign of insole materials or electrode geometry. Filter is obtained directly from the inverse sum of identity matrix and total relation matrix. Because the operator is static, it can be stored in modest memory and executed via a single matrix-vector product at each sampling instant. Computational simplicity makes the method suitable for resource-constrained wearable microcontrollers and allows its addition to existing laboratory or commercial systems already integrating continuous elastomeric interfaces. In addition, directional information contained in the total relation matrix provides physical interpretation of the mechanical network that can guide subsequent optimization of sensor layout or material selection.

Several limitations should be noted. The calibration experiment was conducted under quasi-static, single-point loading conditions corresponding to each sensor individually. During natural walking, contact area changes continuously, and load migrates across the sole; performance of this static operator under dynamic conditions remains to be evaluated. Finally, because validation was limited to a ten-sensor array, extension to denser configurations will require verifying that the spectral radius of the normalized matrix remains below unity to ensure convergence of geometric series.

The framework is useful for researchers who need clearer separation of local and transmitted loads when interpreting experimental plantar pressure data. It assists engineers developing smart insole prototypes who wish to improve measurement fidelity without altering mechanical comfort layers determining wearer acceptance. It also supports clinicians relying on wearable pressure maps for gait evaluation, diabetic foot monitoring, or orthosis assessment, as corrected signals more closely approach actual local pressure distribution under each measurement point. Because correction applies post-acquisition, existing commercial or laboratory systems already including continuous elastomeric interfaces can incorporate operator without hardware modifications, extending the utility of previously manufactured devices.

## 6. Conclusions

The study addressed spatial mechanical interaction occurring between adjacent sensors once integrated into a hybrid footwear structure. Plantar pressure arrays must report local load acting directly above each sensing element, but continuous elastomeric layers providing cushioning also transmit force from one location to another. As a result, the recorded signal contains both local and interference-transferred components, and the reconstructed pressure distribution departs from the real contact pattern. Therefore, the goal was to develop a deterministic framework capable of identifying spatial influence between sensors and compensating for it post-acquisition.

After applying a static decoupling operator derived from DEMATEL total relation matrix, secondary signals narrowed into a narrow residual band between −8 N and 21 N. Corresponding attenuation ratios were between 93%. At the excited sensor in the presented numerical example, the reading dropped only from 1200 N to 1150 N, reduction of approx. 3.7%. Modest change is consistent with model explanation regarding local force absorption in elastomer and should be interpreted as estimated local response, rather than independently verified actual load. Corrected distribution thus recovers spatial picture where dominant contribution remains associated with loaded node, while parasitic contributions from adjacent locations are largely removed.

Normalization ensures spectral radius remains below unity, after which total relation matrix is obtained via standard DEMATEL inversion. Inverting sum of identity matrix and total relation matrix then yields static correction matrix C. Because C is calculated offline and stored as a fixed coefficient array, real-time correction reduces to single matrix-vector multiplication per sampled frame. Computational cost is thus compatible with low-power microcontrollers commonly used in wearable devices.

The approach leaves the physical structure of the insole unaltered. Continuous cushioning layers, rigid structural inserts, and original sensor layout remain exactly as manufactured. Correction occurs entirely post-acquisition. This feature distinguishes methods from strategies relying on dielectric isolation, electrode redesign, mechanical slotting, or adding extra circuit elements. These hardware solutions have also demonstrated substantial cross-talk reductions, though requiring sole or sensor circuit modifications. When comfort, load distribution, or manufacturing constraints prevent such redesign, the post-processing operator provides alternative path mitigating secondary signals.

Beyond numerical correction, the total relation matrix offers spatial description of the mechanical network. Each sensor is characterized by the total influence it exerts on the rest of array and total influence it receives. The resulting cause-effect map identifies dominant transmission pathways and distinguishes nodes acting primarily as dispersion sources from those acting primarily as receivers. Correlation coefficients alone do not provide these directional insights. The map could therefore guide subsequent design decisions related to sensor placement, local stiffening of specific sole regions, or selective introduction of mechanical barriers where strongest transfer pathways are found.

The main contribution of this study is the development of spatial decoupling approach for addressing mechanical cross-talk in plantar pressure sensor arrays. Future work should therefore examine operator under cyclic walking loads, incorporate modest noise term, and test method on higher spatial density arrays and soles made from different elastomer formulations. Sequential excitation of each sensing element would also allow radial completion step to be replaced with fully experimental inputs, eliminating source of model uncertainty. Extending the framework to simultaneous multi-point loading would further clarify if the linear operator remains adequate when multiple sole regions are loaded at same time.

In conclusion, the study demonstrates that spatial filter derived from the DEMATEL total relation matrix can recover substantially clearer representation of local plantar pressure from mechanically coupled sensor arrays. The method preserves the original footwear structure, imposes minimal computational effort, and provides spatial information guiding subsequent design refinement. With future validation under dynamic loading conditions, the approach offers a practical path for improving reliability of wearable plantar pressure measurements, both in laboratory and clinical settings.

## Figures and Tables

**Figure 1:**
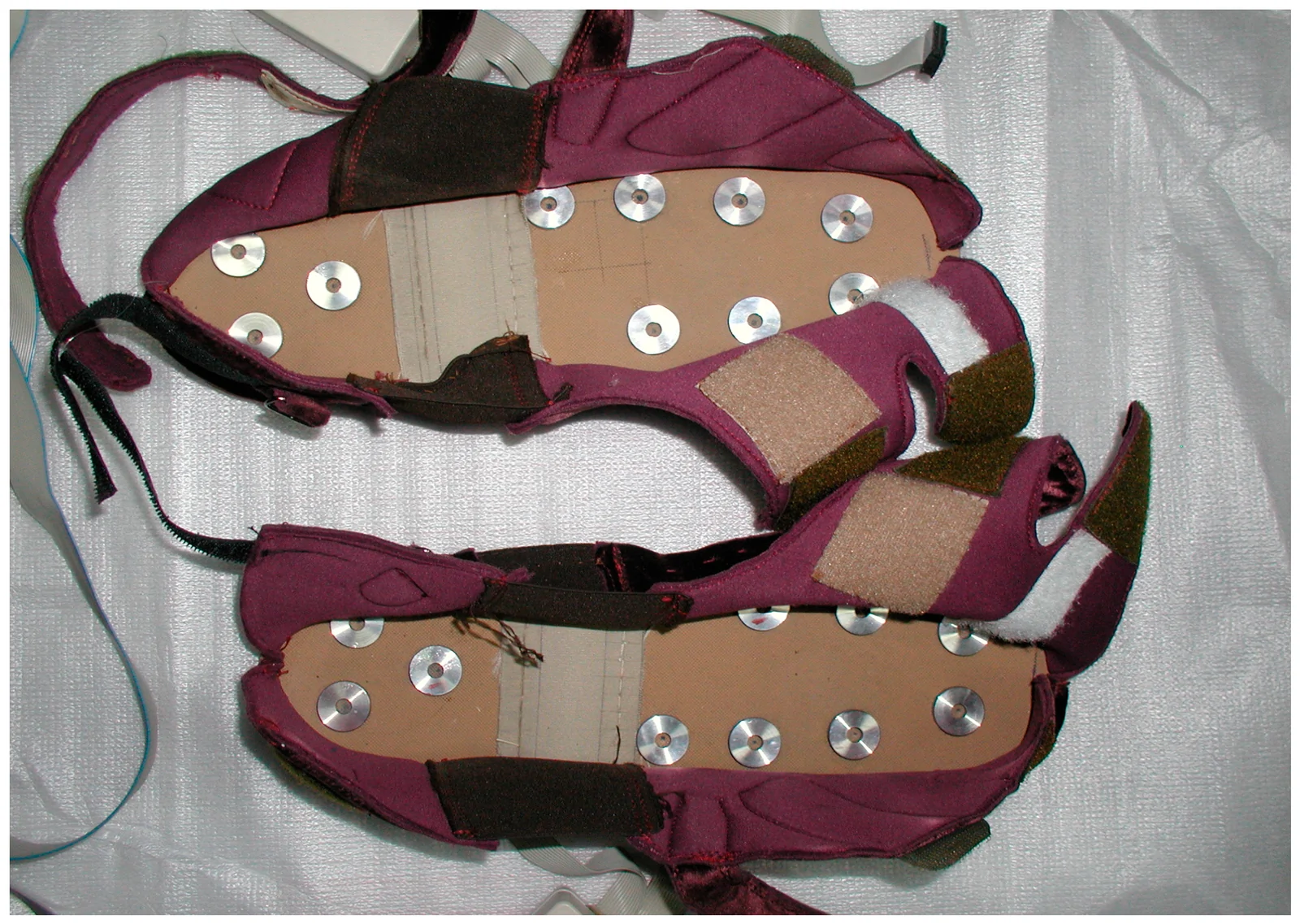
Footwear over-shoe prototype with integrated plantar pressure sensing elements.

**Figure 2:**
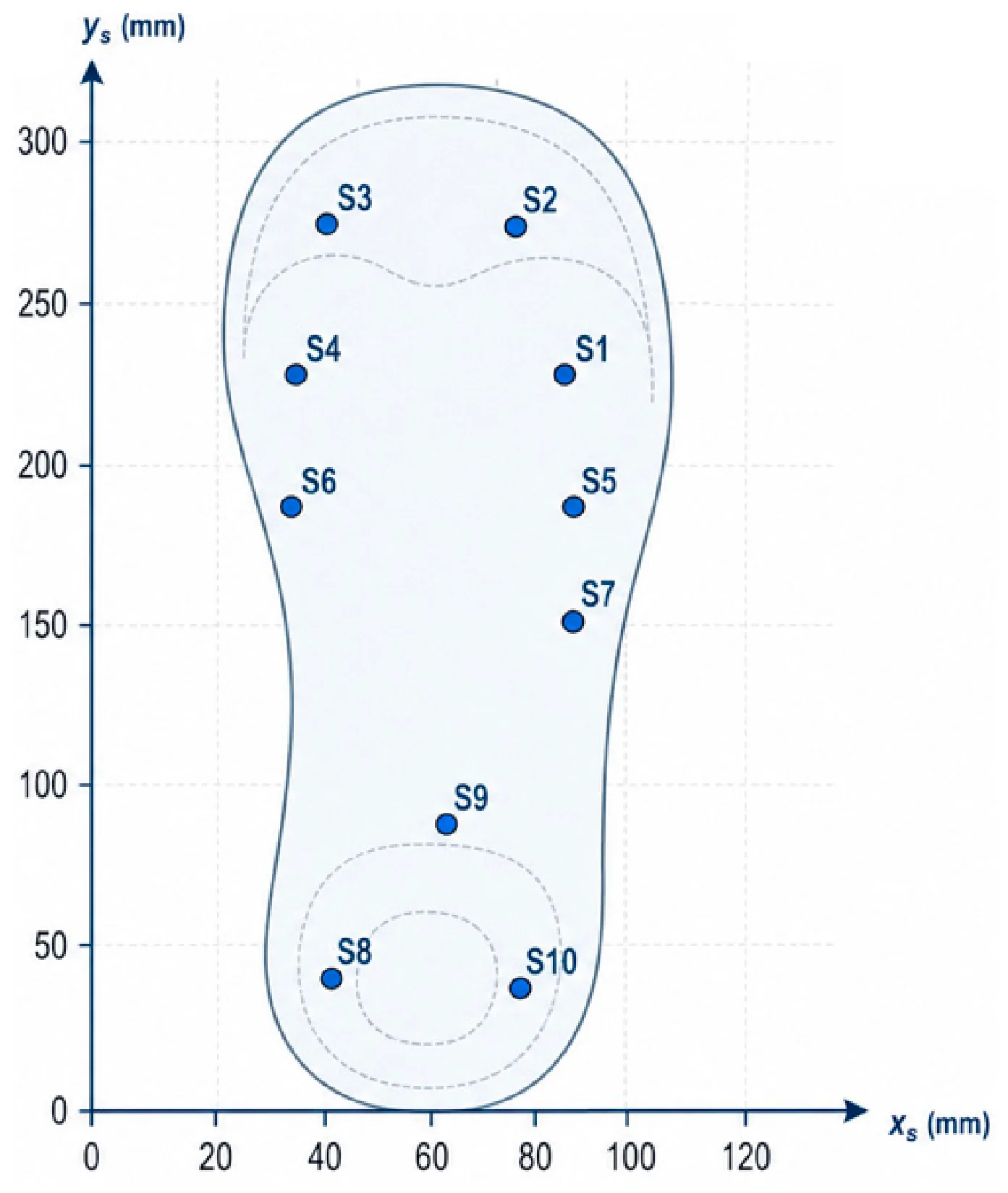
Spatial distribution of the sensing elements on the left-foot support.

**Figure 3:**
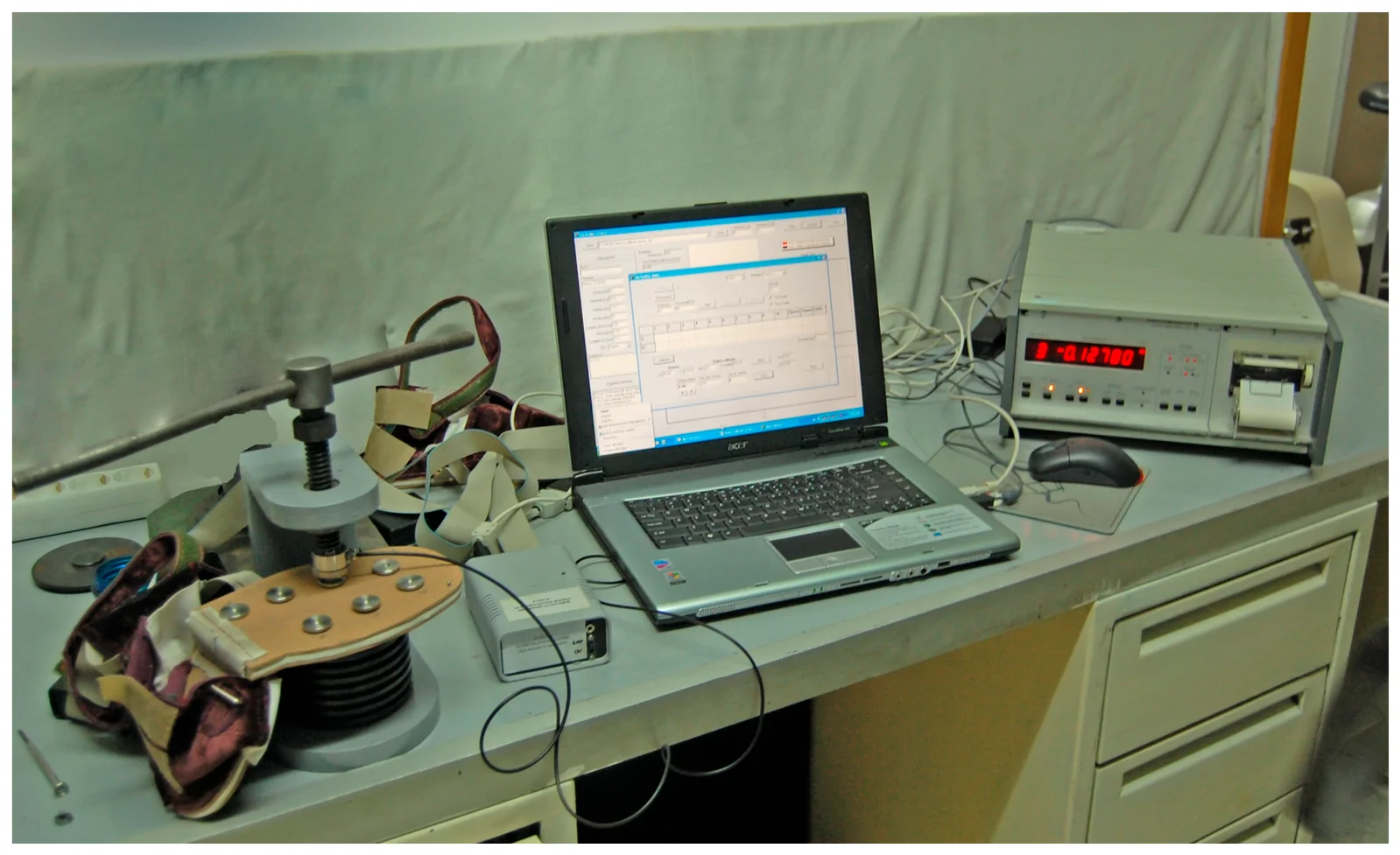
Calibration and data acquisition setup used for controlled loading for all sensors.

**Figure 4:**
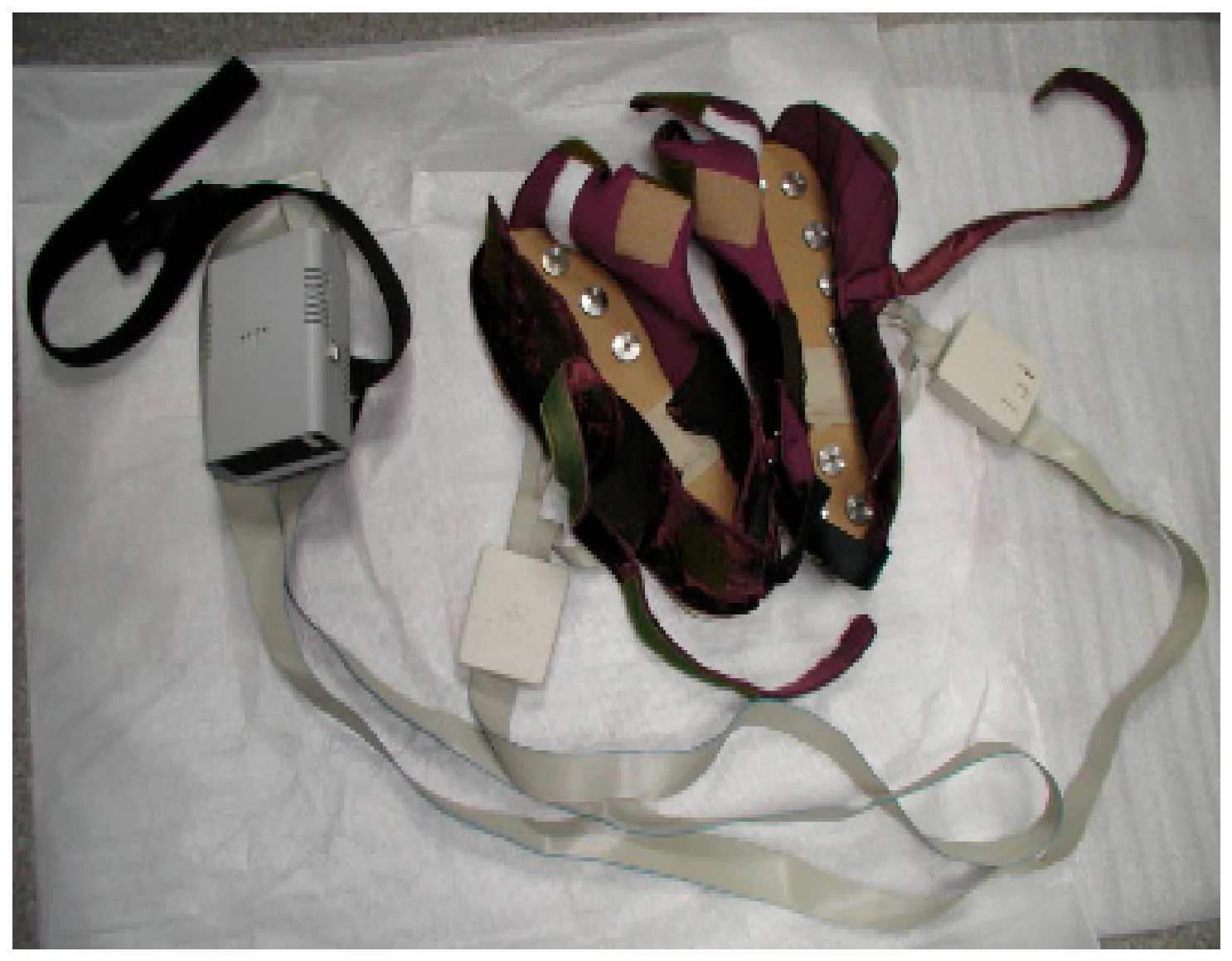
Acquisition module and footwear prototype prepared for sensor readout.

**Figure 5:**
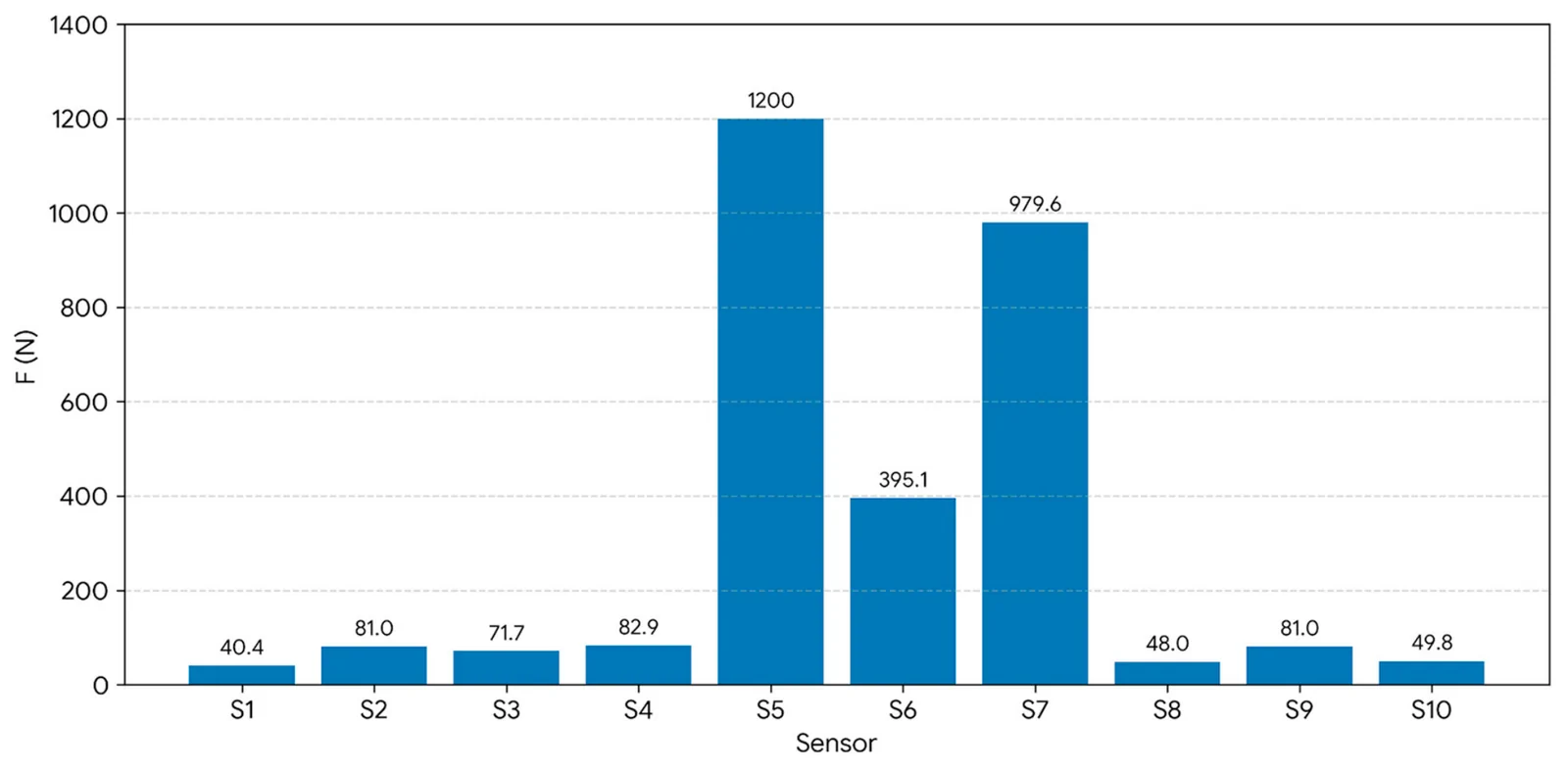
Raw sensor responses recorded during excitation of sensor S_5_.

**Figure 6:**
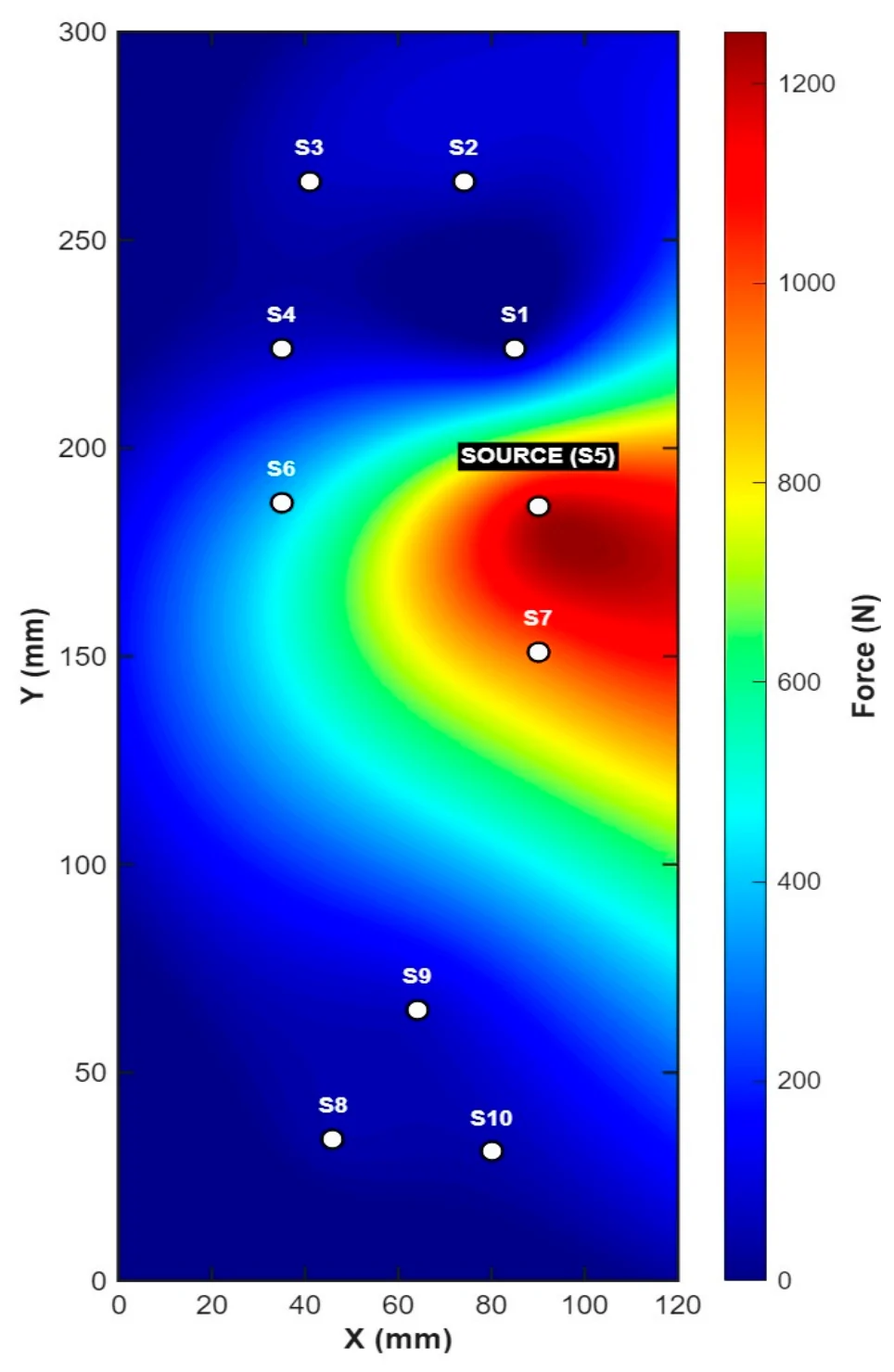
Experimental influence propagation produced by excitation of sensor S5, illustrating the spatial distribution of mechanical cross-talk across the plantar support.

**Figure 7:**
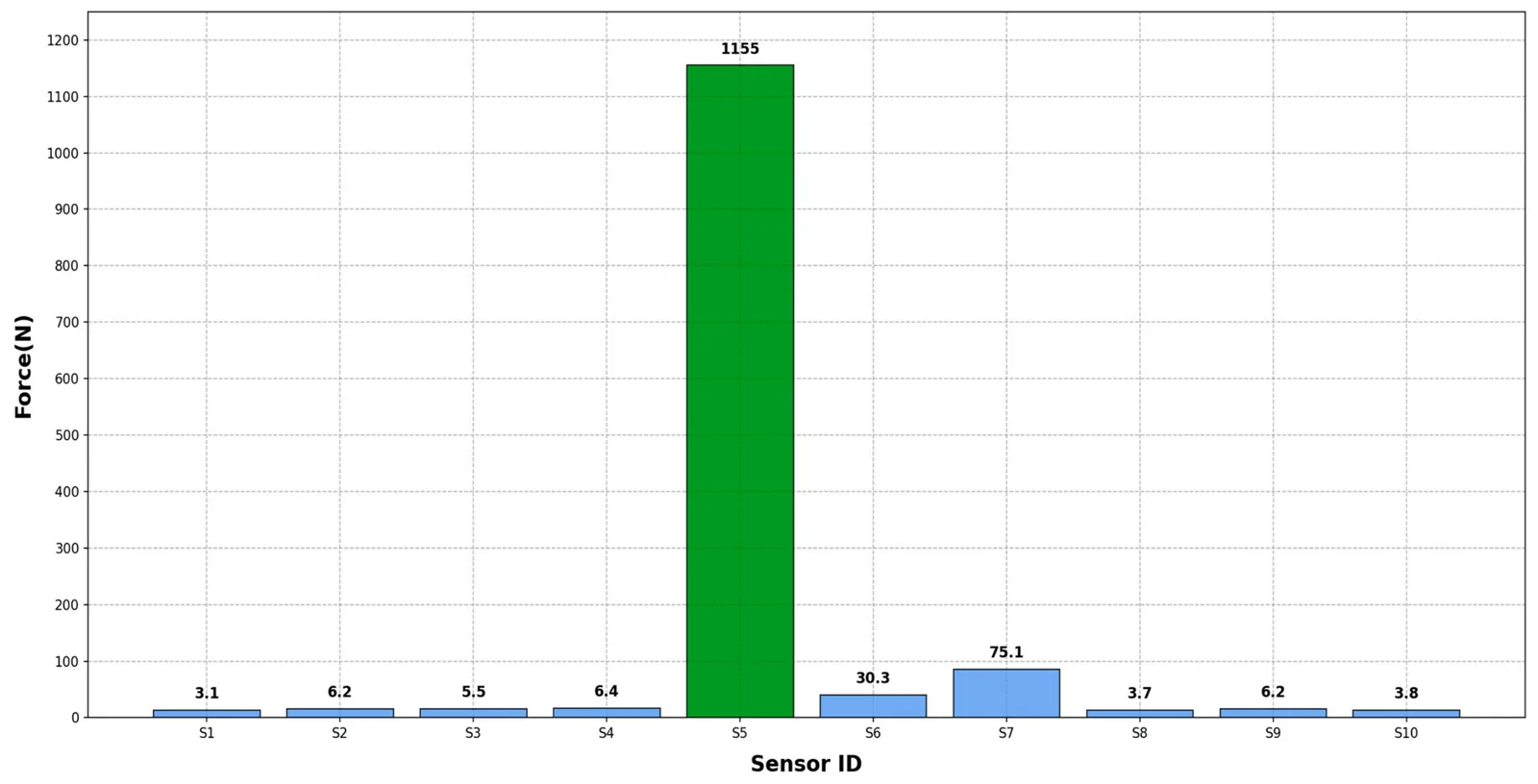
Decoupled force distribution after DEMATEL filter (signal—excitation on S_5_).

**Table 1:** Coordinates of the sensing elements on the left-foot support.

Sensor	*x_s_* (mm)	*y_s_* (mm)
S1	85	224
S2	74	264
S3	41	264
S4	35	224
S5	90	186
S6	35	187
S7	90	151
S8	46	34
S9	64	65
S10	80	31

**Table 2:** Experimental force recorded by each sensor during excitation of Sensor S_5_ location point.

Sensor	*F* (N)
S1	40.4
S2	81.0
S3	71.7
S4	82.9
S5	1200
S6	395.1
S7	979.6
S8	48.0
S9	81.0
S10	49.8

**Table 3:** Direct-relation matrix (*A*).

Factor	S_1_	S_2_	S_3_	S_4_	S_5_	S_6_	S_7_	S_8_	S_9_	S_10_
S_1_	0	47.1	20.2	33.2	40.4	20.3	21.4	1.2	2.3	1.5
S_2_	47.1	0	52.3	26.1	81.0	18.3	7.7	1.0	1.5	1.1
S_3_	20.2	52.3	0	31.5	71.7	8.5	3.7	1.0	1.3	0.9
S_4_	33.2	26.1	31.5	0	82.9	42.7	10.9	1.6	2.3	1.5
S_5_	40.4	81.0	71.7	82.9	0	395.1	979.6	48.0	81.0	49.8
S_6_	20.3	18.3	8.5	42.7	395.1	0	101.6	2.3	4.3	2.7
S_7_	21.4	7.7	3.7	10.9	979.6	101.6	0	3.8	7.2	4.3
S_8_	1.2	1.0	1.0	1.6	48.0	2.3	3.8	0	77.1	102.6
S_9_	2.3	1.5	1.3	2.3	81.0	4.3	7.2	77.1	0	110.3
S_10_	1.5	1.1	0.9	1.5	49.8	2.7	4.3	102.6	110.3	0

**Table 4:** Normalized direct influence matrix (*X*).

Factor	S_1_	S_2_	S_3_	S_4_	S_5_	S_6_	S_7_	S_8_	S_9_	S_10_
S_1_	0	0.025	0.011	0.018	0.022	0.011	0.012	0.001	0.001	0.001
S_2_	0.025	0	0.028	0.014	0.043	0.010	0.004	0.001	0.001	0.001
S_3_	0.011	0.028	0	0.017	0.038	0.005	0.002	0.001	0.001	0.000
S_4_	0.018	0.014	0.017	0	0.044	0.023	0.006	0.001	0.001	0.001
S_5_	0.022	0.043	0.038	0.044	0	0.213	0.528	0.026	0.044	0.027
S_6_	0.011	0.010	0.005	0.023	0.213	0	0.055	0.001	0.002	0.001
S_7_	0.012	0.004	0.002	0.006	0.528	0.055	0	0.002	0.004	0.002
S_8_	0.001	0.001	0.001	0.001	0.026	0.001	0.002	0	0.042	0.055
S_9_	0.001	0.001	0.001	0.001	0.044	0.002	0.004	0.042	0	0.059
S_10_	0.001	0.001	0.000	0.001	0.027	0.001	0.002	0.055	0.059	0

**Table 5:** Total relation matrix (*T*).

Factor	S_1_	S_2_	S_3_	S_4_	S_5_	S_6_	S_7_	S_8_	S_9_	S_10_
S_1_	0.003	0.028	0.013	0.020	0.034	0.019	0.029	0.003	0.004	0.003
S_2_	0.027	0.002	0.029	0.016	0.052	0.015	0.012	0.002	0.002	0.002
S_3_	0.012	0.028	0.001	0.018	0.045	0.009	0.008	0.001	0.002	0.001
S_4_	0.019	0.015	0.017	0.001	0.051	0.026	0.016	0.002	0.002	0.002
S_5_	0.028	0.050	0.042	0.050	0.016	0.240	0.578	0.032	0.051	0.033
S_6_	0.016	0.013	0.007	0.027	0.229	0.016	0.076	0.003	0.005	0.003
S_7_	0.022	0.010	0.005	0.010	0.566	0.071	0.031	0.004	0.007	0.004
S_8_	0.002	0.001	0.001	0.001	0.030	0.002	0.003	0.002	0.047	0.061
S_9_	0.003	0.002	0.001	0.002	0.049	0.004	0.006	0.045	0.003	0.065
S_10_	0.002	0.001	0.001	0.001	0.030	0.002	0.003	0.060	0.065	0.003

**Table 6:** Static decoupling matrix (*C*).

Factor	S_1_	S_2_	S_3_	S_4_	S_5_	S_6_	S_7_	S_8_	S_9_	S_10_
S_1_	0.998	−0.027	−0.012	−0.018	−0.021	−0.006	−0.016	−0.003	−0.003	−0.003
S_2_	−0.026	0.998	−0.028	−0.014	−0.040	−0.003	−0.001	−0.001	−0.001	−0.001
S_3_	−0.011	−0.027	0.999	−0.017	−0.035	−0.001	0.001	0.000	−0.001	0.000
S_4_	−0.018	−0.013	−0.016	1.000	−0.039	−0.012	−0.009	−0.001	−0.001	−0.001
S_5_	−0.022	−0.040	−0.035	−0.039	1.034	−0.134	−0.347	−0.007	−0.016	−0.007
S_6_	−0.011	−0.008	−0.004	−0.021	−0.134	1.012	−0.027	−0.001	−0.002	−0.001
S_7_	−0.012	−0.004	−0.002	−0.006	−0.347	−0.027	1.021	−0.001	−0.002	−0.001
S_8_	−0.001	0.000	0.000	0.000	−0.007	0.000	−0.001	1.002	−0.038	−0.051
S_9_	−0.002	−0.001	0.000	−0.001	−0.016	−0.001	−0.002	−0.038	1.002	−0.054
S_10_	−0.001	−0.001	0.000	−0.001	−0.007	−0.001	−0.001	−0.051	−0.054	1.003

**Table 7:** Comparison between raw and decoupled force signals for S5 used as numerical example.

Sensor Node	Raw Signal (*p*_raw_)	Decoupled Signal (*p*_corrected_)	Cross-Talk Attenuation
S_1_	40.4 N	3.1 N	92.3%
S_2_	81.0 N	6.2 N	92.3%
S_3_	71.7 N	5.5 N	92.3%
S_4_	82.9 N	6.4 N	92.2%
S_5_	1200 N	1155 N	~3.7%
S_6_	395.1 N	30.3 N	92.3%
S_7_	979.6 N	75.1 N	92.3%
S_8_	48.0 N	3.7 N	92.2%
S_9_	81.0 N	6.2 N	92.3%
S_10_	49.8 N	3.8 N	92.3%

**Table 8:** Comparative overview of recent strategies for reducing sensor cross-talk in plantar pressure arrays.

Study	Sensing Principle	Cross-Talk Mitigation Approach	Reported Cross-Talk Performance	Stage of Correction
Dong et al. [10]	Capacitive	Isolated dielectric units	1.30%	Hardware (fabrication)
Zhu et al. [11]	Piezoresistive	Interdigital electrodes + diodes	Essentially free	Hardware (electrode layout)
Wang et al. [9]	Inductive	Circular slots for mechanical isolation	Negligible	Hardware (mechanical design)
Zhang et al. [12]	Piezoresistive	Voltage-mirror multiplexing	Channel independence achieved	Electrical (readout)
Zheng et al. [5]	Triboelectric	Architectural optimization + independence testing	Peak ratio ≈ 15:1	Hardware + testing
Xiong et al. [13]	Resistive laminate	Limited sensor count to reduce lead cross-talk	Not quantified numerically	Design constraint
Present study	Force-sensing array	DEMATEL-derived spatial operator	97.8–99.9% attenuation of secondary signals	Post-processing (software)

## Data Availability

The data that support the findings of this study are available from the corresponding author upon reasonable request.
